# Anemia and associated factors among adolescent girls living in Aw-Barre refugee camp, Somali regional state, Southeast Ethiopia

**DOI:** 10.1371/journal.pone.0205381

**Published:** 2018-10-11

**Authors:** Melaku Tadege Engidaw, Molla Mesele Wassie, Alemayehu Shimeka Teferra

**Affiliations:** 1 Social and Population Health Unit, College of Health Sciences, Debre Tabor University, Debre Tabor, Ethiopia; 2 Department of Human Nutrition, Institute of Public Health, University of Gondar, Gondar, Ethiopia; 3 Department of Epidemiology and Biostatistics, Institute of Public Health, University of Gondar, Gondar, Ethiopia; All India Institute of Medical Sciences, INDIA

## Abstract

**Background:**

Adolescent girls have a higher risk of anemia due to an increased requirement, low intake of hematopoietic nutrients and low intake of a nutrient that enhance absorption of these hematopoietic nutrients. Adolescent girls living in refugee camps are more vulnerable to anemia. The study aimed to determine the prevalence of anemia and associated factors among adolescent girls aged 10–19 years in Aw-Barre refugee camp, Somalia regional state, Southeast Ethiopia.

**Methods:**

A cross-sectional study design was employed. Study participants were recruited using a simple random sampling technique. A structured questionnaire was used to collect the data. Hemoglobin level was tested using HemoCueHb 301 from 10μl finger prick blood samples. Adolescents with a hemoglobin level of <12.5gm/dl after altitude adjustment were classified as anemic. Data were entered using Epi Info version 7.0 and analyzed using SPSS version 20.0. Binary logistic regression was used to explore the association of independent variables with anemia. Variables having P—value ≤ 0.05 was considered to be statistically significant.

**Results:**

Four hundred thirty-seven adolescent girls participated in the study with a response rate of 95.83%. The prevalence of anemia was 22% (95% CI (17.6, 26.1)). Late adolescents were 2 times more likely to have anemia as compared to early adolescents (AOR: 1.95, 95% CI (1.09, 3.47). Those who stayed ≥8 years in the camp were 3 times more likely to develop anemia (AOR: 2.92, 95% CI (1.14, 7.50)). Those who ate heme iron food sources less than one time per month were 11 times more likely to develop anemia compared to those who ate more than twice within a week (AOR: 11.42, 95% CI (3.42, 38.18)).

**Conclusions:**

The prevalence of anemia among adolescent girls was a moderate public health problem. Education and awareness on adolescent nutrition with special attention of late adolescents and duration in the refugee camps is warranted. Moreover, promoting the intake of foods rich in heme iron is suggested.

## Introduction

Anemia is a global public health problem affecting all over the world with major consequences on health, social and economic development. It occurs at all life stages of the human being but is more prevalent in pregnant women and young children [1]. Anemias among adolescent girls happen due to an increased requirement, physical growth, reproductive maturation and cognitive transformation in the continuum of life [2–4]. Even if the cause of Anaemia is multifactorial, globally, the most significant contributor to the onset of anemia is an iron deficiency which attributes at least 50% of the cases of anemia [5]. And other contributing factors were heavy blood loss, parasite infections, acute and chronic infections and presence of other micronutrient deficiencies. All these are very high to be likely in the refugee settings [6].

Anemia is a nutritional disorder mainly caused by iron deficiency especially in disadvantaged adolescent girls [7, 8]. Based on WHO guideline, adolescents are said to be anemic when the hemoglobin level is less than 12mg/dl. But, this might be affected due to the increased iron requirement, decreased iron intake, rapid physical growth, menstrual loss, and high iron demand for hemoglobin (Hgb) formation [9, 10]. Adolescent girls are at higher risk of anemia due to a period of physical growth, reproductive maturation, and cognitive transformations which demands high macro and micronutrients including iron. Adolescent girls in refugee camps are dependent on food aid which leads to irregular eating habits, and intake of low iron content foods as well as taking of nutrients that will inhibit iron absorption [11, 12].

The global prevalence of anemia was 24.8% in (year) and the prevalence was even higher in developing countries (prevalence 40.7%) [6]. Different studies showed that anemia among refugees is a public health problem. The prevalence of anemia among Nepalese refugee and Australia (for newly arriving refugee) was 24% and 16.4% respectively [13, 14]. Among the reproductive age group women of Zaatari Syrian refugee camp in Jordan, the prevalence of anemia was 44.8%. Similarly, 45% of female Iranian refugees older than 10years were anemic [15, 16].

The prevalence of anemia among adolescents in Karen Refugees of Australia, Kakuma refugee camp in Kenya and seven other refugee camps in Nepal was 8.0%, 46%, and 29%, respectively [17, 18]. The magnitude of anemia among food aid beneficiaries’ of refugee girls aged 10–19 years in Fugnido refugee camp, Gambella, Western Ethiopia, was 69.2% [7]. Even if the cause of anemia is multi-dimensional, inadequate dietary intake of micronutrients, especially iron, folic acid, vitamin B-12 due to lack of appropriate complementary foods and high rates of parasitic infections like malaria and HIV were the most common causes of anemia in the refugee settings [7, 19], [20].

From all, 16.4% of the anemia was attributed by the low level of vitamin B-12 deficiency in newly arriving Bhutan, Iran and Afghanistan refugee in Australia and 36% were had low BMI and 6% were had low retinol among Nepalese refugees [14].

The magnitude of being anemic among the rural area of Hassan district, South India was associated with weight loss and low iron status. Among anemic, 60% were underweight, 38% were normal, and 2% were overweight. Only 1% of the adolescent girls were in post-menarcheal status, but 29% of them were found in pre-menarcheal status [21].

In East Africa refugee camps (Kakuma (Kenya), Acholpii (Uganda), Tindouf (Algeria), Fugnido (Ethiopia) and Kebribeya (Ethiopia)), anemia was significantly associated with inadequate intake, malarial infection, and the age of the respondents in each camp [22]. Similarly, the existence of severe anemia and long duration symptoms of Plasmodium Vivax malaria among resettled Eritrean refugees in Sweden were high due to low access to health care during migration or in the area they settled [23].

The highest anemia prevalence (35.3%) was associated with low parental income and being a late adolescent (18.3%) in high schools of Jhaukhel, Nepal adolescent girls [24]. Likewise, being anemic was highly associated with post-menarche (55% and 37%), pre menarche (36% and 17%), and low BMI (48% and 22%) in Kakuma and Nepal refugee camps respectively (23).

Conflict and instability in neighboring countries increase the refugee population in Ethiopia. The numbers of refugees located in Ethiopia were 64,424 in 2008 and 163,650 in 2010. The refugees were mainly from Somalia, Sudan and Eritrea [25, 26] and recently this number increased to 689,107 according to 2015 UNCHR fact sheet. Refugees located in Ethiopia are dependent on food aid (general ration). The general ration contains 16kg of wheat, 1.5kg of Famix, 1.5kg of grain (pulse), 0.9kg of oil, 0.5kg of sugar and 0.5kg of salt per person per month which is low in heme iron and lacks nonheme iron absorption enhancer [26]. Therefore, the aim of this study was to assess the prevalence and associated factors of anemia among adolescent girls aged 10–19 years in Aw-Barre refugee camp, Somalia Regional State, Southeast Ethiopia.

## Methods and materials

### Study design and period

The institutional based cross-sectional study design was employed from March to April 2015.

### Study area

The Aw-Barre refugee camp is found under Eastern Somalia refugee camp coordination office and in the Liben Zone of the regional state. It is located 678km far from Addis Ababa, the capital city of Ethiopia, 78km from Jigjiga (the Town of Ethiopian Somali regional state), and 7km from the border of Somalia. It was established in July 2007 by UNHCR and ARRA of Ethiopia. The camp is located at an altitude of 1621.84 meters (5321feets) above sea level [27]. According to the ARRA report (as of November 2014), the refugee camp had a total population of 12,803. From all 7,382 were female. From all ethnic groups; Hawuyie, Barob, Shekhal, and Bantu account for the highest number of proportion. Among the total population, the numbers of adolescent refugee girls (10 – 19years of age) were 1318.

### Study population

All adolescent girls aged 10–19 years in the Aw-Barre refugee camp was included in this study. Pregnant, breastfeeding and asylum seeker adolescents were excluded from this study due to different iron requirement and difficulty to trace them respectively.

### Sample size

EPI Info 7 Statistical software was used to calculate the number of study participants by taking confidence level of 95% and 5% of marginal error. The overall prevalence of anemia among adolescent refugee girls was 69.2% [7]. And the proportion of anemia among adolescents with low BMI, low household income, being in post-menarche and late adolescents were 60.0%, 35.3%, 55.0% and 18.3% respectively [21, 23, 24]. Based on this information, 380 was the highest sample size after calculating for all proportions by using Epi Info 7 Statcalc software. Then, the final sample size was 456 by adding 20% non-response rate due to an invasive procedure.

### Sampling procedures

From the three refugee camps (namely Aw-Barre, Sheder, and Kebirebeya), Aw-Barre was selected by using the lottery method. There were a total of 1,318 adolescent girls aged 10–19 years in Aw-Barre refugee camp. During the study, a simple random sampling technique was applied to select the required study participants in this camp. To set the sampling frame, recently updated (revalidated) lists of adolescent refugee girls with respective house number were obtained from the administration of refugee and returnee affairs bureau, refugee database manager’s office. After the random selection, the data were collected from house to house.

### Data collection method and equipment

A modified and pretested UNHCR standardized expanded nutrition survey (SENS) questionnaire was used to collect the data. The questionnaire had questions related to sociodemography, health status, hemoglobin, nutritional status, diet frequency and type, other food and income source beyond the general ration. This questionnaire had both open and close-ended questions.

The English version of the questionnaire was translated into the local language (Somali) and it was translated back into English by the third person to check its consistency.

Two days training was given to the basic skills of measuring hemoglobin, calibration of instruments, interview techniques, ways of obtaining the written consent or assent and how to interact with respondents as precautions for data collectors and supervisors. The data collectors were nurses and medical laboratory technicians under the supervision of two field supervisors.

Before starting data collection, always we checked materials and equipment like weight scale with height stand, HemoCuvettes, HemoCueHb 301 machine, and sanitary materials like cotton, alcohol, and glove.

A finger prick was done after wiping the finger with alcohol soaked cotton for hygiene and safety measures, and then the pricked finger was gently pressed to get a sample of 10μl blood. The sampled blood had been put on HemoCuvettes and to be inserted into the HemoCueHb 301 machine. Finally, the hemoglobin level was read and recorded on the questionnaire. Weights and heights were measured using a weight scale and height stand machine by placing the participants into Frankfurt position respectively. Then, the result of weight and height were recorded to the nearest 0.1kg and 0.1cm respectively.

### Data quality assurance

The pretest was done outside of the study area (in the Sheder refugee camp) on 20 samples before the actual data collection period. The weight scale was calibrated by using 1kg standard weight, height measurements were checked with other meter taps and the HemoCueHb 301 machine was calibrated with the 3 calibrating Hemo–solutions (EurotolHb 301 control solution which is a bovine-based solution). The pricing and taking of sampled blood were taken after drying a weep finger by avoiding of squeezing the finger to avoiding air bubbles during filling of HemoCuvettes.

During the data collection time, communication between the data collectors, supervisors and the principal investigator were held on a daily basis to discuss the progress and problems faced during the data collection time.

The collected data were checked for completeness and consistency by the supervisors and the principal investigator during and after data collection.

A definition of concepts and terms had been done clearly with the Somali language to avoid ambiguity. The data collectors and supervisors were recruited outside of the refugee health center to avoid information bias related to familiarization with respondents. The data were managed by editing, verification, coding, classification, and tabulation of collected data during data entry and analysis.

### Operational definitions

**Anemia**: when the adolescent girl had a hemoglobin level below 12.5 mg/dl after adjusting for an altitude (>1000m) by adding 0.5%g/dl [8] and further classified as mild (11–11.9 g/dl), moderate (8.0–10.9g/dl) and severely (lower than 8.0g/dl) anemic based on the hemoglobin level [9].

**Poor nutritional status**: if the BMI was <18.5kg/m^2^ and classified as severe, moderate and mild if the BMI is ≤16kg/m^2^, 16 – 17kg/m^2^ and 17–18.4kg/m^2^ respectively [28].

**Abnormal menstruation**: If the girl reports the experience of vaginal bleeding two or more times per a month, the amount of blood is higher when she compared to the previous usual and having a recent history of abortion or miscarriage [5].

**Stunting**: if the height for age Z score was below -2SD from the median value of WHO reference data [29].

**Thinness/Wasting**: If the BMI-for-age Z score was less than −2SD from the median value of WHO reference data [29].

**Dietary diversity score**: was the qualitative measure of food consumption that reflects household access to the variety of foods which is categorized as low dietary diversity (≤3 food groups), medium dietary diversity (4 and 5 food groups) and high dietary diversity (≥6 food groups) [30].

### Data processing and analysis

The collected data were checked for completeness, then compiled, coded and finally entered to Epi Info 7 and then exported to SPSS version 20 Software for analysis. Also, WHO Antthroplus software was used to calculate the Z–score of adolescent nutritional status.

Descriptive analysis was carried out to describe the variables. A binary logistic regression model was used to assess the independent effects of each variable towards anemia. Those variables with a p-value of 0.2 and below during bi-variable analysis were fitted into a multivariable logistic regression model to identify the independent contribution of each variable.

Variables having a p-value of less than or equal to 0.05 was considered as statistically significant for the development of anemia after fitting into multivariable logistic regression. Odds ratio with a 95% confidence interval was done. The Hosmer-Lemeshow goodness of fit test was performed to assess how the constructed model was good (p-value = 0.79).

### Ethical consideration

Ethical clearance was approved and obtained from the Institutional Review Board of the University of Gondar College of Medicine and Health Science, Institute of Public Health. An official letter was also obtained from the administration of the refugee and returnee affairs of Ethiopia and letters were prepared for the local authority of the selected refugee camp. Written informed consent and or assent were obtained from each study participant after they were included in this study. The assent was taken in front of the mother and/or caregiver and both of them were agreed and signed on the consent form after detail explanation of the procedure and the research. The purpose of the study was explained to study participants before giving consent. We deliver information on the rights to interrupt the interview and refuse to give a blood sample. We said to them, the sampled blood was used only to measure the Hgb level in front of the study participants and after measuring and recording the result, the sample was immediately discarded into a disposal safety box. Confidentiality was maintained at all levels of the study. Adolescent girls with anemia were referred to the refugee health center by using refugee health center internal referral slip.

## Results

### Socio-demographic characteristics of respondents

From the total sample size, 437 adolescent girls participated in the study with a response rate of 95.83%. The mean age ± SD of participants was 13.96 ± 2.70years. Sixty percent of the participants were found between the ages of 10 to 14 years. One-fourth of participants was from Hawuyie clans and followed by Bantu (17.8%). Half of the households in the camp had a family size of 5–9 persons per household. More than ninety-three percent (407) of the respondents stayed in the camp over seven years [**Table 1**].

**Table 1:** Socio-demographic characteristics of respondents in Aw-Barre Somalia refugee camp, Southeast Ethiopia, 2015 (n = 437).

Variables		Number	Percentage (%)
Age	10–14	262	60.0
	15–19	175	40.0
Marital status	Single	414	94.7
	Married	23	5.3
Ethnicity	Hawuyie	111	25.4
	Asharafa	40	9.2
	Bantu	78	17.8
	Dir	49	11.2
	Darod	29	6.6
	*Others	130	29.8
Family size	1–4	31	7.1
	5–9	231	52.9
	10–19	175	40.0
Educational status	Do not to read & write	45	10.3
	Able to read &write	17	3.9
	Grade 1–8	327	74.8
	Grade 9–12	46	10.5
	College and above	2	0.5
Have domestic animals	Yes	14	3.2
	No	423	96.8
Selling of food aid items	Yes	428	97.9
	No	9	2.1

*Others ethnicity: Gore, Gaboye/Maddagan, Arab, Shikal, Tumal, Samaran, Isak, Barbo, Rahawayan, Shanshi, Geladi, Moreshe, Jalele and Durukbo

### Meal frequency

From all, those who ate 2 times per day was 19 (4.3%) and three and four times were 410 (93.88%) and 8 (1.82%) respectively. More than half of the respondents consume heme rich food sources less than once per a month and the more than 90% were less than once per a month consume fruits and vegetables as shown in Table 2 below (**Table 2**).

**Table 2:** Meal frequency related characteristics of respondents in Aw-Barre Somalia refugee camp, South East Ethiopia, 2015.

Variables	Categories	Frequency	Percentage
Heme iron food sources	> twice a week	60	13.7
	once per a month	269	61.6
	< one time per month	108	24.7
Fruits and vegetables	> twice per week	8	1.8
	Once per month	34	7.8
	< one time per month	395	90.4
Egg	> twice per a week	45	10.3
	Once per month	254	58.1
	< one time per month	138	31.6
Milk and milk products	Once in a day	6	1.4
	Once per week	6	1.4
	< than per week	425	97.3
Tea consumption	>2 times in a day	27	6.2
	once in a day	387	88.6
	> once per week	23	5.3

### Dietary diversity

From the total, all of them were used rice, spaghetti, and macaroni as a staple diet of the family. The major source of family food was general ration which is distributed and donated by ARRA and WFP respectively. Most of the household 428 (97.8%) sold the distributed general ration to buy other foodstuffs and the most common item to be sold was wheat.

In this study, 294 (67.3%) of them had a good diversity score (≥6 food items). Households with medium and poor DDS were 122 (27.9%) and 21 (4.8%) respectively. But, the variety of the food items in each category was very low throughout the households due to the general ration.

### Diseases and menstruation-related conditions

Even if we apply different probing techniques, respondents did not report any known chronic diseases. Only 01 (0.2%) of the respondent had diarrhea in the past 2 weeks. From all, 256 (56.8%) of them were found at post-menarche at the mean age of 13.96 years. Among girls with a menstruation cycle, only 5 (0.5%) of them had abnormal menstruation.

### Nutritional status and hemoglobin level

The mean weight ± SD of respondents was 43.39kg ± 11.00kg. Also, the mean height and hemoglobin ± SD were 152.35cm ± 9.59cm and 13.47g/dl ± 1.50g/dl respectively. The mean BMI ± SD was 18.47kg/m^2^ ± 3.58kg/m^2^. Based on WHO BIM classification, the rate of severe, moderate and mild malnutrition was 114 (26.1%), 59 (13.5%) and 70 (16.0%) respectively. On the other hand, the prevalence of overweight and obesity were 16 (3.7%) and 4 (0.9%) respectively.

As compared to WHO growth reference curve of height for age, 0.9% and 7.1% of them were <-3SD (severely stunted) and <-2SD (moderately stunted) respectively with mean ± SD of -0.45 ±1.12. The wasting rate by using their BMI for age was 13.7% (1.6% <-3SD, 12.1% <-2SD, 9.8% <+1SD, 1.8% <+2SD, 0.2% <+3SD) with a mean ± SD of -0.52 ± 1.21.

Before determining the individual anemic status, the cutoff point for Hgb was adjusted for altitude. The altitude of Aw-Barre refugee camp is 1621.84 meters above sea level. Hemoglobin cutoff value became 12.5g/dl after adjustment for the altitude (+0.5g/dl) [8]. Based on this, the overall prevalence of anemia among adolescent refugee girls was 22% (95% CI (17.6, 26.1)). Among anemic adolescent girls, 3 (0.7%), 36 (8.2%) and 57 (13.1%) were severe, moderate and mild anemia respectively.

### Associated factors of anemia

A crude analysis was done to assess the existence of an association between the independent variables and anemia. In binary logistic regression; age, family size, duration stayed in the camp, current marital status, the frequency of heme iron-rich foods consumption and frequency of egg consumption were found to be positively associated variables with the anemic status of adolescent girls.

After fitting these significant variables in multivariable logistic regressions; age, length of duration stayed in camp, intake of egg and intake of heme iron-rich foods were independently associated with the development of anemia. Late adolescents were 2 times more likely to have anemia as compared to early adolescents (AOR: 1.95, 95% CI (1.09, 3.47). Those who had a higher length of time in the camp were 3 times more likely to have anemia than those who stayed short duration (AOR: 2.92, 95% CI (1.14, 7.50)).

Those who ate heme iron-rich foods less than one time per a month was 11 times more likely to develop anemia as compared to those who ate more than twice within a week (AOR: 11.42, 95% CI (3.42, 38.18)). Adolescents used eggs more than twice per a week were 68% (AOR: 0.32, 95% CI (0.11, 0.95)) less likely to develop anemia as compared to once per month user as shown in **Table 3**.

**Table 3:** Binary and multivariable logistic regression result of associated factors with anemia among adolescent refugee girls of Aw-Barre Somalia refugee camp, South East Ethiopia, 2015.

Variables	Category	Anemia		Crude OR (95% CI)	Adjusted OR (95% CI)
		Yes	No		
Age in years	10–14	52	210	1	1
	15–19	44	131	1.36 (0.86, 2.14)	1.95(1.09, 3.47)*
Marital status	Single	88	326	1	1
	Married	8	15	1.98 (0.81, 4.81)	0.61(0.20, 1.89)
Family size (median)	< 9	37	175	1	1
	≥ 9	59	166	1.68 (1.06, 2.67)	1.22 (0.71, 2.11)
Duration in the camp (median)	<8	6	68	1	1
	≥ 8	90	273	3.74 (1.57, 8.90)	2.92 (1.14, 7.50)*
Heme iron rich foods	> twice in a week	9	51	1	1
	once per a month	24	245	0.56(0.24, 1.27)	1.05(0.36, 3.06)
	< one time per month	69	39	10.03(4.46, 22.54)	11.42(3.42, 38.18)*
The frequency of eating egg	> twice per week	12	33	1	1
	Once per month	23	231	2.14(0.49, 9.41)	0.32 (0.11, 0.95)*
	< one time per month	71	67	22.78(5.31, 97.77)	0.75 (0.23, 2.47)

*statistically significant at α = 0.05, 1 = reference group

## Discussion

This study shows that the prevalence of anemia among adolescent refugee girls was 22% (95% CI, (17.6, 26.1). According to the WHO classification, it is a moderate public health problem. The finding of this study is very low when compared with the findings of the study (62.9%—severe public health problem) in Fugnido refugee camp (Ethiopia) [7]. This might be due to the difference in sample size, refugees in Fugnido camp and assessment time and study populations with dissimilar local circumstances.

The prevalence of anemia in this study is low due to the absence of infectious and communicable diseases, but in Kakuma refugee camp (Kenya) the prevalence was 46% due to the high rate of infectious and communicable diseases [23]. This might relate to the high burden of infectious diseases due to demography variation among adolescent with inadequate health care coverage in a refugee setting.

The overall low Hgb results showed a variation of anemic status by age, duration stays in the camp, intake of egg and meat and intake of heme iron-rich food sources).

Late adolescents were more significantly affected by anemia in this study. The odds of developing anemia were 2 times more among late adolescents as compared to early adolescents (10 – 14yrs) (AOR: 1.95, 95% CI (1.09, 3.47). This finding is similar to a study done on Nepal [14]. However, age was not significantly associated with anemia in the study done in a stable population of Hassan district of India [21]. As the result, it indicates older girls are more risk because of a growth spurt and menstruation. The possibility of rapid growth in stature, muscle mass, and fat mass during adolescence time leads to a greater daily requirement of iron and other micronutrients.

The duration stayed in the camp was also one of the significant variables that increase the development of anemia in adolescent girls. Those adolescents stayed ≥ 8yrs were 3.12 times more likely to develop anemia (AOR: 2.92, 95% CI (1.14, 7.50). This finding is supported by a similar study in Ethiopia among long-term North and East Africa refugees based on their dependency on general food aid [7, 19]. This might be due to the fact that as age increases the requirement increase and the stored micronutrient became depleted, also not replaced by what they were eating, the chance to involve in work is high and may lead to the highest demand of energy.

Having anemia among more than twice per a week users were 68% (AOR: 0.32, 95%CI (0.11, 0.95)) less likely as compared to once per a month users and those who use heme iron food sources less than one time per a month was 11.42 (AOR: 11.42, 95%CI (3.42, 38.18) times more likely to develop anaemia as compared to those who frequently use. This finding is in agreement with a survey on refugees of North and East Africa bases at Kakuma (Kenya), Acholpii (Uganda), Tindouf (Algeria), Fugnido (Ethiopia) and Kebribeya (Ethiopia) which anemia was associated with inadequate intake iron-rich foods [19]. This might be due to general ration dependency among refugee which led to having a limited dietary micronutrient supply, unable to meet their demand, lacks Heme iron (meat and egg) food source and potential enhancers for micronutrient absorption.

The study was limited in linking anemia to specific micronutrient deficiency, unable to establish any possible causal link to the specific micronutrients deficiency as well as to know the specific food groups which favor this anemic condition. This study did not include clinical assessment of diseases, type of anemia and clinical assessment of anemia.

## Conclusions

The prevalence of anemia among adolescent refugee girls was a moderate public health problem, and a small proportion of the adolescent girls had severe anemia. Ages, length of stay in camp, low intake of egg and heme rich food sources were the predictors for the development of anemia among refugee adolescent girls.

Based on the finding, UNCHR/WFP/ARRA will consider increasing the intake of heme iron food sources and vegetable gardening in order to enhance iron reach food intake in the households by taking the best practices of the hosting community.

Also, the ongoing practice of conducting annual surveys of stakeholders (UNCHR/WFP/ARRA) among food aid beneficiaries of adolescent girls at least by using anthropometric and Hgb is important to monitor the prevalence.

A health professional should consider providing health education and counseling based on age and menarche status.

The high level of anemia had seen in long-term refugees argues in favor of further enhancements in food aid fortification or the option of cash transfer and the strengthening of nutrition and public health programs.

## Supporting information

S1 File Data of refugee adolescent anemia. (SAV)

## Abbreviations

AIDS: Acquired Immune Deficiency Syndrome
ARRA: Administration of Refugee and Returnee Affairs
BMI: Body Mass Index
CDC: Communicable Diseases Control
DDS: Dietary Diversity Score
EDHS: Ethiopia Demographic and Health Survey
Hgb: Hemoglobin
HIV: Human Immunodeficiency Virus
SPSS: Statistical Package for Social Science
UNHCR: United Nation Higher Commission for Refugee
UNFPA: United Nation Fund of Population Agency
WFP: World Food Program
WHO: World Health Organization
